# The impact of leisure time physical activity on mental health and health perception among people with cancer

**DOI:** 10.34172/hpp.2020.19

**Published:** 2020-03-30

**Authors:** Jaehyun Kim, Junhyoung Kim, Areum Han

**Affiliations:** ^1^Department of Health and Human Performance, Texas State University, San Marcos, TX, USA; ^2^School of Public Health, Indiana University, Bloomington, IN, USA; ^3^Department of Physical Education, Dongduk Women’s University, Seoul, South Korea

**Keywords:** Oncology, Leisure activities, Mental health, Health status, Health promotion

## Abstract

**Background:** People with cancer often report high levels of negative psychological symptoms and poor perception of health due to cancer treatment and activity limitations. Prior studies have suggested that participation in leisure time physical activity (LTPA) can reduce negative psychological symptoms and improve health perception. However, a few studies have examined the contribution of LTPA to health benefits among people with cancer. Thus, we aimed to examine how a different level of LTPA engagement contributed to mental health and health perceptions among people with cancer.

**Methods:** Using the 2017 Health Information National Trends Survey (HINTS) data, cross sectional data of 504 respondents diagnosed with any types of 22 cancers listed in the survey questionnaire were analyzed. A multivariate analysis of variance (MANOVA) was used to test for mean differences in mental health and health perception among the three different LTPA groups(i.e., inactive, moderately active, and vigorously active groups).

**Results:** Results indicated that people with cancer who reported higher levels of LTPA scored higher on mental health and health perception than those with lower levels of LTPA (Pillai’s trace= 0.060, F (4,944) = 15.06, P < 0.001).

**Conclusion:** This finding suggests that individuals with cancer gained more health benefits through high engagement in LTPA. Moreover, we suggested that LTPA can be used as an important therapeutic intervention to promote health quality and wellbeing among people with cancer. Implications for practical suggestions are further discussed.

## Introduction

Cancer has become a global public health concern, as it is the leading or second leading cause of death in many countries.^1^ According to the International Agency for Research on Cancer (IARC), in 2018, 17 million people were diagnosed with cancer worldwide, and it is expected to grow to 27.5 million people by 2040.^2^ As the number of people diagnosed with cancer has continued to rise, public health researchers have gained more interest in health promotion and quality of life for people with cancer. They have been attempting to create a variety of programs to promote health and wellbeing among people with cancer.

Prior research has suggested that people with cancer tend to experience negative psychological symptoms, such as fear, anxiety, and depression.^3,4^ Due to negative psychological symptoms, people with cancer have reported poorer health perception and diminished quality of life.^5,6^ For example, Eng and colleagues^7^ found that nearly 40% of breast cancer patients showed poor self-rated health. They suggest that promoting health perception and quality of life among people with cancer is imperative. Thus, it is important for healthcare providers to design and implement health promotion interventions conducive to promoting quality of life and wellbeing for people with cancer.

Healthcare providers have proposed that participation in leisure-time physical activity (LTPA) is a non-pharmacological health promotion intervention, as it increases quality of life and health among people with cancer. Previous studies have provided evidence that LTPA participation contributes to social and psychological health among cancer survivors.^8-10^ These studies have indicated that LTPA serves as an important vehicle for improving health status and overall quality of life among people with cancer. In addition, multiple studies have focused on investigating the effects of physical exercise interventions on psychosocial functioning.^11,12^ They concluded that physical exercise interventions reduced negative psychological symptoms, such as depression, anxiety, and stress among people with cancer.

In spite of the importance of LTPA benefits among people with cancer, previous studies have mainly focused on understanding the role of LTPA as a cancer prevention strategy.^13,14^ Those studies indicated that participation in LTPA was associated with a decreased risk of developing cancer. From health promotion perspectives, little research exists on examining the contribution of LTPA to mental health and perceived health among people who have been diagnosed with cancer. In addition, while multiple studies have found the positive association of LTPA participation with health of people with cancer,^15,16^ only a few studies have investigated how different levels of LTPA participation contributed to mental health.^17,18^ That is, the association of non-participation in LTPA with health perception and mental health among people with cancer is currently unknown. Therefore, the purpose of this study was to examine how different levels of LTPA engagement contributed to mental health and health perceptions among people with cancer. Specifically, we aimed to test whether there are significant mean differences in mental health and health perception across the three different LTPA groups (i.e., inactive, moderately active, and vigorously active groups).

### Leisure time physical activity, health perception, and mental health

Previous studies have explored the relationship between LTPA and health perceptions among people with cancer.^19,20^ They found that people with cancer who actively participated in LTPA reported higher health perceptions than those who did not. According to Schootman et al,^21^increased participation in LTPA was associated with a reduction of fair-poor self-rated health during both the first year after diagnosis and one year later. In addition, Lee et al^22^ found that older adult cancer survivors who engaged in LTPA reported better health status. Moreover, previous studies suggested that LTPA serves an important non-pharmacologic means to improve overall health-related quality of life among people with cancer.^10,17^ For example, Paxton et al10 found that childhood cancer survivors actively involved in LTPA increased overall health-related quality of life, including physical, social, cognitive, and psychological functions.

Empirical studies have emphasized LTPA interventions for mental health promotion among people with cancer.^9,23^ Kolden et al^24^ examined the effect of 16-week group exercise programs (e.g., aerobic and resistance training) on mental health among people with breast cancer. They found that participation in group exercise programs helped participants reduce depressive symptoms and improve overall quality of life. In addition, after completion of 10-week group exercise programs, such as gymnastics, relaxation, walking, and jogging, breast cancer patients reported reduced anxiety and depression and improved maximal oxygen uptake and body image.^25^ In the recent study, Ho et al^26^ examined relationships among fatigue, physical activity, depressive symptoms, and quality of life among childhood cancer survivors. They found participation in LTPA helped alleviate feelings of fatigue and depressive symptoms and ultimately promoted the perceived quality of life.

Several studies have sought to investigate the relationship between levels of LTPA and mental health among people with cancer. For example, a population-based study of breast cancer patients found that participation in low- and moderate-intensity LTPA was associated with better mental health (i.e., low levels of depression).^27^ Furthermore, Lee et al^22^ found the older adult cancer survivors who participated in vigorous LTPA showed significantly higher levels of health status than those who engaged in low-intensity LTPA. These studies suggest that greater involvement in LTPA is positively associated with mental health among people with cancer. Rogers and colleagues^28^ focused on rural breast cancer survivors and revealed that those who met physical activity guidelines (i.e., ≥ 500 MET-mins/week) showed significantly lower scores on depression than those who reported physical activity levels less than 500 MET-min/week. Although the authors provided insights into the possible relationship between sitting behaviors and depression levels among cancer survivors, they did not consider those who did not report LTPA (i.e., LTPA non-participants). Therefore, the present study examined how a different level of LTPA engagement (i.e., inactive, moderately active, and vigorously active groups) contributed to mental health and health perceptions among people with cancer.

## Materials and Methods

### Data source

This study used the 2017 Health Information National Trends Survey (HINTS), a nationally represented survey of non-institutionalized adults in the United States. The HINTS includes a wide range of health information, such as cancer prevalence, health status, and health-related behaviors, as well as public use of health-related information. The 2017 HINTS conducted random-digit dialing telephone surveys to provide a nationally represented sample of U.S. households. Telephone interviews were conducted with one adult from each household. The HINTS sampling method and survey design have been presented in detail elsewhere (Nelson et al).^29^ The 2017 HINTS had a sample of 3285.

For the purpose of this study, we extracted 504 respondents who had been diagnosed with any type of 22 cancers listed in the survey questionnaire. The most frequently reported cancer was skin cancer (n = 162), followed by breast cancer (n = 106) and prostate cancer (n = 56). Descriptive statistics (Table 1) indicated that the sample was comprised of 303 female participants (61.8%), and 187 male participants (38.2%), and the mean age was 67 years. The majority of respondents were non-Hispanic White (75.7%). The most common educational level was “Some college” (30.2%), followed by “High school graduate” (24%) and “A Bachelor’s degree” (22%).

### Measures

*Demographics*

This study included age (years), gender, education, and race/ethnicity as demographic variables. Educational attainment included five categories: Less than high school, high school graduate, some college, bachelor’s degree, and post-baccalaureate degree. There were five categories of race/ethnicity: White, Black, Hispanic, Asian, and other.

*Independent variable*

Leisure time physical activity (LTPA) was assessed by asking respondents to indicate the number of days and the duration of the moderate-intensity physical activities they undertake in a typical week. Moderate-intensity physical activity was considered as 4.0 MET (the metabolic equivalent of task).^30^ Continuous scores for LTPA were calculated as follows: MET level (4) × minutes of activity per day × days per week. That is, cancer survivors who meet the LTPA guideline (i.e., 4 METs for 150 minutes per week) refer to those who reported more than 600 MET-minutes per week. For this study, cancer survivors were categorized into three different LTPA groups: (1) inactive group (those who did not report any LTPA; 0 MET-min/week), (2) moderately active group (those who reported less than 600 MET-min/week), (3) vigorously active group (those who reported more than 600 MET-min/week).

*Dependent variables*

Health perception was measured with one question: “In general, would you say your health is...” This item was assessed on a 4-point Likert-type scale (1 = Excellent, 2 = Very Good, 3 = Fair, and 4 = Poor) and was reverse coded for our data analysis. Thus, higher scores indicate better health perception among cancer survivors. Mental health was measured with four items. That is, respondents were asked to indicate how often those problems (i.e., little interest or pleasure, hopelessness, nervousness, and worrying) have bothered them over the past two weeks. An example of items includes “Past 2 weeks, how often were you bothered by feeling nervous, anxious or on edge?” The items were assessed on a 4-point Likert-type scale (1 = Nearly every day, 2 = More than half the days, 3 = Several days, and 4 = Not at all). Scores on the four mental health indicators were averaged. Thus, cancer survivors with higher scores indicate those who reported better mental health.

### Data analysis

All analyses were conducted using SPSS version 24.0 (SPSS Inc, Chicago, Illinois). Descriptive analysis was used to identify cancer patients’ characteristics with regard to demographics and study constructs. We found that the outcome variables of interest (i.e., mental health and health perception) were moderately correlated (r = 0.38) and, thus, decided to use a multivariate analysis of variance (MANOVA) to test for differences in mental health and health perception among the three different LTPA groups. When significant differences occurred between groups in MANOVA tests, Scheffe post-hoc analyses were conducted to determine which means significantly differ from each other. Pillai’s Trace test was used as the assumption of homogeneity of covariance across the groups was violated.^31^

## Results

### LPTA groups and health perception and mental health

Descriptive statistics (Table 2) indicated that the vigorously active group had the highest scores on mental health and health perception, followed by moderately active and inactive groups. In our MANOVA tests, a statistical test for equality of covariance matrices was found to be significant (Box’s M = 12.944, F = 2.144, *P* < 0.05). Since the assumption of homogeneity of covariance across the groups was violated, we used Pillai’s trace as a test statistic. Results of the MANOVA (Table 3) indicated that there are statistically significant differences in mental health and health perception (Pillai’s trace = 0.060, F (4,944) = 15.06, *P* < 0.001). The univariate tests (Table 4) revealed a statistically significant difference in the mean mental health among the three LTPA groups (F (2,472) = 10.20, *P* < 0.001). That is, cancer patients who report higher levels of LTPA reported better mental health than those with lower levels of LTPA. The univariate tests also indicated a statistically significant difference in the mean health perception among the three LTPA groups (F (2,472) = 29.84, *P* < 0.001). This shows that cancer patients who report higher levels of LTPA reported better health perception than those with lower levels of LTPA. Scheffe post-hoc test (Table 4) indicated that mean scores on mental health were significantly lower in the inactive group than either moderately active or vigorously active groups, while no significant difference was found between moderately active or vigorously active groups. With regard to health perception, Scheffe post-hoc test revealed that the vigorously active groups had significantly higher scores than the other two groups, and the moderately active group had significantly higher scores than the inactive group (Table 4).

## Discussion

This study examined how a different level of LTPA engagement contributed to mental health and health perceptions among people with cancer. The results of this study found that a higher level of LTPA participation led to better mental health and health perception. This study indicates that people with cancer gain more health benefits through high engagement in LTPA. This study also suggests that LTPA can be used as an important therapeutic intervention to promote health quality and wellbeing among people with cancer.

Previous studies have found that greater LTPA participation resulted in better mental health among people with cancer.^24,25,27^ The results of the current study expand the body of literature, suggesting that vigorous engagement in LTPA leads to better mental health compared to groups with lower levels of LTPA engagement.^32^ For example, Rogers et al28 found that cancer survivors who met physical activity guidelines scored significantly lower on depressive symptoms than those who did not meet the guideline. Consistent with this study, the current study also revealed that people with cancer who actively participated in LTPA can gain mental health benefits. In addition, it is noteworthy that mean scores on mental health were significantly lower in the inactive group than either moderately active or vigorously active groups. This finding may suggest that engaging in at least some LTPA is better than non-participation with regard to mental health among people with cancer.

Results of our study indicated that participants with higher levels of LTPA engagement reported higher scores on health perception than those with lower levels of LTPA engagement. The findings of this study supported previous literature that suggests higher levels of LTPA participation increased health perception and health-related quality of life.^12,18,22^ The current study suggests that increased LTPA participation may allow participants to increase their perceptions on health and wellbeing while enhancing health status. Moreover, researchers have provided evidence that health perception and health-related quality of life are associated with mortality.^33,34^ For example, Vejen et al^35^ found that high levels of self-rated health and health-related quality of life were all associated with decreased mortality in intensive care unit survivors. Given this, our finding has a significant implication that it is important for health care professionals to develop intervention programs conducive to promoting LTPA, which in turn may improve health perception and reduce cancer mortality.

The majority of studies have focused on the role of LTPA as cancer prevention (e.g., Eliassen et al^36^). These studies demonstrated that regular LTPA involvement reduced the risk of developing cancer across a wide range of the population. Along with the benefits of LTPA as prevention, this study extends the importance of LTPA participation as a health promotion intervention. In particular, higher levels of LTPA engagement serve as an important health promotion strategy for people with cancer to increase health perception and mental health. Thus, this study extends the body of research on cancer studies to include that increased participation in LTPA positively influences healthy behaviors and health promotion.

This study has several limitations. In the sample used for this study, the majority of people with cancer were non-Hispanic Whites (75.7%). Therefore, the findings of this study may not be generalizable across diverse ethnic groups. Future research could extend our analysis by using a sample equally distributed across different ethnic groups. In addition, future studies could examine whether there are ethnic differences in LTPA, health perception, and mental health in a sample of people with cancer.

Another study limitation we found was the self-reported LTPA data. Although the HINTS used a validated assessment of physical activity, we acknowledge that several biases (e.g., recall bias) may exist. In addition to the amount of LTPA (i.e., MET-min/week), it would be interesting if future research examines the contributions of different types of leisure activities (e.g., outdoor leisure versus indoor leisure) to health perception and mental health among people with cancer.

Last, this study did not differentiate the current status of cancer, such as cancer patients and cancer survivors. Cancer patients’ LTPA involvement can be different from cancer survivors. If future studies investigate a group difference on mental health and health quality through a level of LTPA, they can provide insightful information and knowledge to healthcare providers and therapists.

## Implications and Conclusion

The ACS’s physical activity guideline recommends that people with cancer should participate in “at least 150 minutes a week of moderate-intensity, or 75 minutes a week of vigorous-intensity aerobic physical activity, or an equivalent combination of moderate- and vigorous intensity aerobic activity”.^37^ Despite the health benefits of LTPA participation, most people with cancer did not meet the physical activity recommendation.^38^ These previous studies suggest that physical inactivity is predominant among cancer survivors and demonstrate a critical need for developing interventions to encourage their LTPA participation.

One health promotion strategy is to provide leisure education programs to people with cancer. Bullock and Mahon^39^ defined leisure education as ‘‘an individualized and contextualized process through which a person develops an understanding of self and leisure education and identifies and learns the cluster of skills necessary to participate in freely chosen activities that lead to an optimally satisfying life’’. Healthcare providers and therapists working with people with cancer need to design and offer leisure education programs in which people with cancer can obtain specific information about community-based physical activity/exercise programs or information about LTPA-related community resources. Through these educational programs, people with cancer can pursue active leisure lifestyles, engage in LTPA, gain resources and LTPA skills and techniques, and develop positive social interactions.

Previous research has stressed the importance of social support as a means to facilitate leisure participation and reduce negative psychological symptoms among people with cancer.^40,41^ Given the benefits of social support, healthcare providers and therapists create social support groups related to group exercise programs that encourage LTPA participation for people with cancer. For example, the creation of community-based programs, such as natural walking groups, can be beneficial for people with cancer, who are physically inactive. In addition, by participating in group-based exercise programs with others who are experiencing cancer, they can build unique friendships, bond as a group, and encourage LTPA participation for each other.

This study examined the differences in the mean values of health perception and mental health among three different LTPA groups of people with cancer. We found that people with cancer in the vigorously active group reported higher scores on both health perception and mental health than those with lower levels of LTPA groups. This study extends the body of knowledge that higher engagement in LTPA can result in better health perceptions and mental health among people with cancer. By creating and implementing a variety of physical activity/exercise programs, people with cancer can be physically active in their leisure time and, thus, improve their health-related quality of life.

## Ethical approval

Not applicable.

## Competing interests

The authors declared no potential conflicts of interest.

## Funding

No funding was received for this study.

## Authors’ contributions

The first author (JK) proposed a research idea and write on the sections of Introduction and Literature Review. Then, the first author had worked on analyzing the collected data with the second author (JHK) and third author (AH). JHK as corresponding author was responsible for writing Material and Methods and Results sections. We as a research team worked together to write on the sections of Discussion and Implications and Conclusion. Each author has contributed to the editing process throughout the whole manuscript. Finally, all authors have approved the manuscript for submission to the *Health Promotion Perspectives.*

**Table 1:** Demographic characteristics of individuals with cancer

**Gender**	**N**	**Valid %**
Female	303	61.8
Male	187	38.2
**Age (years)**	**Mean**	**Standard Deviation**
	67	12
**Education**	**N**	**Valid %**
Less than high school	30	6.2
High school graduate	117	24.0
Some college	147	30.2
Bachelor's degree	107	22.0
Post-baccalaureate degree	86	17.6
**Race/ethnicity**	**N**	**Valid %**
White	340	75.7
Black	51	11.4
Hispanic	36	8.0
Asian	5	1.1
Other	17	3.8

**Table 2:** Descriptive Statistics on Mental Health and Health Perception

	**Levels of LTPA**	**Mental Health**		**Health Perception**	
		**Mean**	**Standard Deviation**	**Mean**	**Standard Deviation**
LPTA groups	Inactive (163)	3.28	0.774	2.78	0.023
	Moderately active (153)	3.56	0.599	3.31	0.927
	Vigorously active (159)	3.59	0.668	3.58	0.983
	Total	3.47	0.699	3.22	1.000

**Table 3:** Multivariate tests for mental health and health perception by levels of LTPA

**Effect**		**Value**	**F**	**Hypothesis** ***df***	**Error** ***df***	***P*** **value**	**Partial Eta squared**
Intercept	Pillai’s trace	0.966	6786.633	2.000	471.000	<0.001***	0.944
LTPA levels	Pillai’s trace	0.120	15.061	4.000	944.000	<0.001***	0.060

*P < 0.05, **P < 0.01, ***P < 0.001.

**Table 4:** Significant Univariate Effects and Post-Hoc Test for Levels of LTPA

**Dependent Value**		***df***	**Mean square**	**F**	***P*** **value**	**Partial Eta squared**	**Scheffe** ^a^
Mental Health	9.592	2	4.796	10.204	0.000***	0.041	2, 1 > 0
Mental Perception	53.287	2	26.643	29.844	0.000***	0.112	2, 1 > 0 2 > 0

*P < 0.05, **P < 0.01, ***P < 0.001. ^a^ 0 = non-LPTA group, 1 = low LTPA group, and 2 = high LTPA group.
